# The Clinical Society of Maryland

**Published:** 1901-02

**Authors:** 


### *SOCIETY REPORTS.*

---

### THE CLINICAL SOCIETY OF MARYLAND.

BALTIMORE, November 2, 1900.

The meeting was called to order by the President, Dr. W. J. Todd, who, in assuming his new duties, delivered a brief address.

### A CASE OF ACROMEGALY. EXHIBITION OF PATIENT. DR. J. HALL PLEASANTS.

Dr. Pleasants exhibited a colored man, the subject of acromegaly, associated with giantism. The man was of enormous size, the hands and feet particularly showing huge development. For years he had been unable to secure shoes large enough to fit him. All the usual signs of acromegaly were present. Skiagraphic pictures of the long bones and of the bones of the hands and feet were exhibited showing very well the peculiarities belonging to this disease.

The doctor referred to the literature upon this subject, calling especial attention to the possible relationship between acromegaly and giantism, and briefly considered the treatment of this affection.

#### DISCUSSION.

*Dr. Osler:* I have been very much interested in the treatment of acromegaly, having had some four or five cases here, three of which were under observation for a prolonged period of time. All of them received very thorough and rigid treatment with thyroid extract without any of them receiving the slightest benefit. One patient lost a little weight at first under the use of the drug, just as any patient treated with thyroid will, but in one case, the well-known "Pauline," the patient actually throve on the extract. Two of our cases took pituitaria also, without any benefit.

There is one very typical case of this disease in this city, a colored woman, who has not a very large frame, but whose face and hands are perfectly characteristic. I have seen her several times on the street cars.

It is a very remarkable disease, and I am inclined to hold with Marie in the non-union of giantism with acromegaly. The matter is still under observation and discussion, however. I might mention an interesting historical point in reference to the disease in this country. I was on the staff of the *Medical News*, when Marie's report came out, and Dr. Hays sent me the paper with the request for an abstract and an editorial. The following week I went to Toronto, and one of the physicians asked me to visit a remarkable case he had in the hospital. I went with him and saw a perfectly typical case o f acromegaly. It was one of the two first cases reported in this country. The other was a merchant, who had a very beautiful wife, and they came to be known as the "beauty and the beast."

*Dr. Randolph:* These cases usually show a primary optic nerve atrophy in the later stages, and in the case to which Dr. Osler has referred, there was a very singular sector-like defect in the field. It might be interesting to have such an examination made here, if it has not been done.

*Dr. Pleasants:* I asked Dr. Friedenwald to-day to examine the eye, and he attempted to do so, but the patient would not submit to an examination at the time, and we had to desist.

*Dr. Osler:* Dr. Randolph's remarks remind me that it was an ophthalmologist who first made the diagnosis, in one of these cases, basing his opinion upon the peculiar appearance of the field.

*Dr. Paton:* In this connection I would like to remark that there has been a few cases of enlargement of the pituitary body without acromegaly. To-night, before coming here, I tried to find some sections of this kind that I have in my possession. Undoubtedly, in all the cases of acromegaly, with reliable observations, changes in the pituitary body have been found, but there are a great many changes in the pituitary body without symptoms of acromegaly, so that the hypothesis used to explain the latter affection is not as strong now as it was a few years ago.

A series of reports on the men and papers at the International Medical Congress, held in Paris, August, 1900, were then presented. The first, dealing with the section on otology, was presented by Dr. J. J. Carroll.

The section of otology in the International Medical Congress was presided over by Dr. E. Gelle, and included in its membership many well-known men, such as Politzer, Hartmann, Dundas, Grant, Pritchard, Meniere, and others. The average number attending the daily sessions was about sixty or seventy. While the United States had her full quota of doctors at the Congress, many of her best otologists were not present. The entire program consisted of about fifty-seven papers, not one of which was written by a delegate from the States. In the absence of Dr. Katz of Berlin, an American, acting as his substitute, exhibited some transparent microscopic preparations of the middle and inner ear. He was the only American to respond to a number on the program.

The subjects discussed covered pretty well the entire field of otology. Besides individual papers, reports were made upon important topics as follows: 1. Upon a project to make the recording of hearing tests uniform. Drs. Schiffers of Liege, and Hartmann of Berlin. 2. Upon hearing exercises in the treatment of deaf mutes. Dr. Urbantschitsch of Vienna, and Schwendt of Basle. 3. Surgical treatment of sclerosis of the middle ear. Drs. Siebenmann of Basle, and Botey of Barcelona. 4. On otitic pyemia. Drs. Grant of London, and Brieger of Breslau. 5. On the causes and treatment of the vertigo of Meniere. Drs. Pritchard of London, and Moll of Arnheim. 6. On toxic diseases of the labyrinth. Drs. Gradenigo of Turin, and Easpariantz of Moscow.

The paper which probably represented the hardest work and elicited the liveliest interest was the report upon hearing exercises in the treatment of deaf mutes. The subject was not an unfamiliar one to the members of the Congress, some of them recalling, perhaps, the experiments of Ernaud in 1761, of Pereire in 1768, and of Itard in 1802, while among the more modern workers in this line, they would have thought of Toynbee, Jager, Gallaudet and Javal. But no doubt, the one who has of recent years labored most over the method and brought it most prominently before the world is Urbantschitsch, one of the two men who prepared the report.

In 1888 he tried the exercises on a deaf and dumb boy, who could hear only letters when shouted very loudly in the ear. In the course of the experiments, the boy gradually became able to hear spoken sentences at from two to four feet, and finally, at the end of two years, he was able to follow a class in one of the common schools. This observation prompted him to give the matter greater attention, and his subsequent efforts have been rewarded with even better results. The method is about as follows:

In a great many deaf mutes, who are thought to be totally deaf, there is a remnant of hearing power capable of being developed, and this may be brought about by loud sounds produced either by the voice or by suitable instruments. Let us suppose, for example, a patient who cannot hear loud conversation nor the sounds of the tuning fork. In order to detect the amount of hearing left, a vowel like A or O is shouted repeatedly into one ear. If no response is given, a funnel is made of the hands so as to intensify the sound and the different vowels repeated in a loud voice close to the ear. If still no response, loud notes of the accordion are used to awaken, as it were, the dormant powers of the labyrinth. An interesting experiment by Urbantschitsch may serve as an illustration. The patient was a 22-year-old deaf mute, who, after repeated exercises with the vowels, was able to tell the difference between them with the right ear; the left ear, however, showed only the slightest trace of hearing. He then supplemented the exercise by the use of the accordion as follows: While shouting the vowel A in the left ear (worse ear) and at the same moment sounding the corresponding note of the accordion in the right ear, an effect upon the auditory apparatus of the left ear was at once apparent. By repeated tests, he was convinced that it was not the passing of the sensation upon the right ear to the left, because as soon as the calling of the vowel was discontinued, although the accordion was still being sounded in the right ear, every trace of sensation in the left ear immediately disappeared. From this and other observations Urbantschitsch maintains that auditory sensations, present in the perceiving apparatus of one ear, can excite the acoustic centres of the other, therefore by making the two ears hear, the better ear may help the worse, but the converse does not hold good, namely, that the worse ear may help the better.

When the ear has been aroused to hear the loud notes of the accordion and has learned to appreciate the different vowels, the exercise is continued with consonants, then, according to the progress made, with words, phrases and sentences. Hearing in deaf mutes seems at times to be favorably affected by more or less continuous sound, as is shown by a deaf man who gradually regained his hearing by methodical exercise. This patient told Dr. Urbantschitsch that when he spoke out loud he could not at first hear anything, but after a few minutes of continuous loud talking, he could discern, first, letters, then syllables, words and finally whole sentences.

The difficulties met with in this method are numerous. The exercises themselves are apt to become tiresome to the children and monotonous to the teachers. The results are gained very, very slowly, and only after months of constant application for the pupil and with a rare amount of patience on the part of the instructors. On account of these difficulties, this system has been confined to institutions for the care and education of deaf mutes. For very small children, still too young to enter such institutions, the nursery should be supplied with a goodly number of wind and stringed musical instruments, music boxes, bells, etc. For children about three or four years of age, a fair supply of picture books should be on hand and the daily lesson should be the calling aloud the name of something in the book or around the room, while at the same time pointing to the object itself. After six years of age the regular hearing exercises may be systematically carried out. The importance of having intelligent, faithful, patient, persevering teachers cannot be exaggerated. They should engage and keep the interest of their unfortunate pupils in the work and should be quick to see just how much exercise an ear can stand, for when the ear becomes fatigued by such exercise, the hearing power rapidly diminishes and the lesson should be immediately interrupted and not resumed until the next day.

This method of treatment for deaf mutes is not without its opponents. Politzer and others who have examined the pathological anatomy of such cases contend that hearing exercises can have no effect upon a labyrinth destroyed almost entirely by a morbid process. Bezold, while appreciating the argument of the opponents, is, nevertheless, in favor of the method, and claims that it can produce an increase in the perceptive power, and a development of those parts of the labyrinth which have remained more or less unaltered by disease. He advocates the hearing exercises only for those deaf mutes who learn to hear sounds after a certain number of exercises, and he thinks that in institutions there should be two groups of pupils, one capable of being taught by this method and the other to remain under the older methods.

The consensus of opinion among the otologists of the Congress was, as far as I could judge, that where the deafness is total or nearly so, it is useless to employ the hearing exercises, but that those subjects who possess an appreciable amount of audition should have the hearing exercises, and, as helping agents, the phonograph and musical instruments which have agreeable tones such as the piano, organ, accordion, etc.

If we consider the practical value of this method by improving the modulation of the voice, by giving deaf mutes a form of language, by throwing around them a safeguard in their ability to hear sounds of approaching danger, especially in the crowded streets of large cities and by enabling them to understand spoken language, I think we may with reason consider the adoption of the same system for the benefit of the 41,000 deaf mutes in our own country.

SECTION ON GYNECOLOGY. DR. T. S. CULLEN.

In the gynecological section there was not much which was absolutely new. Most of the prominent and best known representatives from the continental countries were present. The principal papers dealt with the conservative treatment of myomata. They seem to be adopting the conservative plan of myomectomy in place of hysterectomy more and more.

Cancer of the uterus was considered but the discussion turned principally on the question of treatment. Orth recommended the vaginal hysterectomy for nearly all cases. It was my pleasure to follow, him and I advocated the abdominal route. When the general discussion was taken up I believe almost all of the ten or twelve speakers expressed a preference for the abdominal route, the belief being that this gave the best results.

Considerable attention was given to the treatment of extrauterine pregnancy. In America this condition is, I think, observed rather earlier than elsewhere because the general practitioners recognize it more quickly and in consequence more lives are saved.

In Paris a great deal of study has been devoted to the introduction of cocaine into the spinal column for the production of anesthesia in abdominal operations. I saw a partial removal of a sarcoma, the treatment of an umbilical hernia and a case of pus tubes with inflammation of the uterus in which a complete hysterectomy was performed under cocaine. The patient talked to the observers during the time of operation. I think the view of the Americans present was that its application should be restricted to those cases in which we dare not give a general anesthetic. We do not know what the after effects of the use of cocaine may be.

I think the Clinical Society of Maryland may feel that it has been highly complimented in that two of its members, Drs. Osler and Jacobs, were selected as the two executive officers from the United States.

SECTION OF DERMATOLOGY. DR. T. C. GILCHRIST.

One feature of the dermatological division was a separate room where many photographs, paintings, models, etc., were shown and stereopticon views of transparencies were given. Several important subjects were put forward for general discussion and in accordance with an idea suggested by Brock a resume of the papers to be read was printed and distributed among the members so that one could have an idea of the points to be discussed.

The principal subject of discussion, perhaps, was the question "Is eczema a parasitic disease?" Some years ago an investigator found a certain cocci present in a number of cases and stated that he could reproduce the disease from these. His views were received with favor by some, with doubt by others, and the result of the discussion at the Congress seemed to be that it was not proven as yet that it is a parasitic disease. The monococcus referred to proved to be the epidermidus albus of Welch. The vesicle of eczema is probably sterile.

Another topic of discussion was as to the parasitic natnre of slopechia areata. A French observer has thought he had discovered the organism of this disease. Here again, however, the consensus of opinion seemed to be that it is probably a parasitic disease but the organism is not yet proven.

The next subject discussed was tuberculosis of the skin, not only the varieties in which the presence of the bacillus could be shown but also those in which there was a strong hereditary history without the demonstrable evidence of the presence of the organism.

An excellent feature of the Congress was the regular morning exhibition of patients, 50 or 60 being shown each day. The most prominent feature of the whole Congress was a consideration of lupus vulgaris. Fourteen cases were sent down from Copenhagen to show the results of Finsen's treatment, and these results were wonderful; the cosmetic effects were such that in most of them you would not have suspected they had ever had the disease. That sunburn of the skin is due to the ultra violet rays of light has been proven. Finsen demonstrated that if a tube of muriate of silver be put under the skin of a dog and rays of light made to penetrate the skin, the silver will be found blackened when removed. It was next learned that if the blood be pressed out of the skin first, the rays of light penetrate it much more rapidly, in about one-third of the time. Finsen's method of treatment consists practically in concentrating the sun's rays, minus the heat rays, on the parts, and this is done for an hour every day for from six to twelve months. His apparatus consists of a plano-convex lens of two pieces, the space between being filled with a solution of ammonium sulphate. A similar apparatus is pressed closely against the part of the body to be treated to produce anemia and the tubes attached to this are so arranged as to carry a continuous current of cold water and thus prevent burning of the skin from overheating of the apparatus. The sun not shining constantly in Copenhagen, Finsen has used the electric light of about 30 amperes, concentrating it by a telescopic arrangement. At the Santorni Hospital I saw them treating cases, three or four at a time, and the results were really beautiful.

Adjourned. H. C. REIK, M.D., Secretary,
5 W. Preston St., Baltimore.

